# Overexpression of LT, an Oncoprotein Derived from the Polyomavirus SV40, Promotes Somatic Embryogenesis in Cotton

**DOI:** 10.3390/genes13050853

**Published:** 2022-05-11

**Authors:** Chao Lu, Yunxiao Wei, Zhigang Meng, Yongming Liu, Abid Muhammad Ali, Qinfei Liu, Mubashir Abbas, Yanan Wang, Chengzhen Liang, Yuan Wang, Rui Zhang

**Affiliations:** 1 Biotechnology Research Institute, Chinese Academy of Agricultural Sciences, Zhongguancun, Nandajie No. 12, Haidian District, Beijing 100081, China; bararayung123@163.com (C.L.); weiyunxiao@caas.cn (Y.W.); mengzhigang@caas.cn (Z.M.); yongming.liu@zskybio.com (Y.L.); abid@caas.cn (A.M.A.); a824922953@163.com (Q.L.); mubashirabbas3164@yahoo.com (M.A.); wangyanan@caas.cn (Y.W.); liangchengzhen@caas.cn (C.L.); wangyuan07@caas.cn (Y.W.); 2 College of Life Sciences, Inner Mongolia University, University West Street No. 235, Saihan District, Hohhot 010000, China

**Keywords:** upland cotton, *LT* gene, NEC (non-embryonic callus), EC (embryogenic callus), somatic embryogenesis

## Abstract

Although genetic transformation has opened up a new era for cotton molecular breeding, it still suffers from the limitation problem of long transformation periods, which slows down the generation of new cotton germplasms. In this study, *LT* gene (SV40 large T antigen), which promotes the transformation efficiency of animal cells, was codon-optimized. Its overexpression vector was transformed into cotton. It was observed that EC (embryogenic callus) formation period was 33% shorter and transformation efficiency was slightly higher in the LT T0 generation than that of control. RNA-seq data of NEC (non-embryonic callus) and EC from LT and control revealed that more DEGs (differential expression genes) in NEC were identified than that of EC, indicating *LT* mainly functioned in NEC. Further KEGG, GO, and transcription factor analyses showed that DEGs were significantly enriched in brassinosteroid biosynthesis pathways and that bHLH, MYB, and AP2/ERF were the top three gene families, which are involved in EC formation. In addition, the key genes related to the auxin pathway were differentially expressed only in *LT* overexpression NEC, which caused early response, biosynthesis, and transportation of the hormone, resulting in EC earlier formation. In summary, the results demonstrated that *LT* can promote somatic embryogenesis in cotton, which provides a new strategy for improving cotton transformation and shortening EC formation time.

## 1. Introduction

A variety of plant explants can induce somatic embryogenesis through in vitro culture. The induction of somatic embryogenesis in vitro is considered to be the direct evidence of plant cell totipotency [1]. Callus could be induced from pericyclic cell, stem cells, and young embryonic epidermis by exogenous hormone treatment [2]. Subsequently, non-embryogenic cells transdifferentiate into tightly formed embryogenic cells inside and outside the callus mass. The single or multiple embryogenic cells re-differentiate into somatic embryos in the absence of hormones [3]. Recent studies have found the conversion of somatic cell to embryonic cell is preconditioned by three factors, namely regulatory factors to derepress the inhibition of embryonic determinants, the expression of embryonic characteristic genes, and the induction of auxin [4]. Somatic embryogenesis is also mainly determined by three factors, including the developmental period of explants, the induction of hormones, and the expression of embryonic transcription factors [5]. In addition, the somatic cells can also be induced by ectopic expression of embryonic regulators such as *WUS*, *LEC1*, *LEC2*, etc. [6].

Hypocotyl is widely used as an explant in agrobacterium-mediated genetic transformation in cotton. Based on this method, a number of cotton varieties with abiotic and biotic resistance have been cultivated, opening up a brand-new era for cotton molecular breeding. With the widespread application of gene editing technology, the demand for cotton transformation continues to expand [1]. However, genotype dependence and long transformation periods severely impede the wide application of agrobacterium-mediated transformation in cotton. The long transformation period greatly delays functional genomic study in cotton, which in turn hinders the breeding of new varieties. To tackle this challenge, numerous studies have been conducted to explore the molecular mechanism underpinning somatic embryogenesis in cotton. Most of the studies focused on the identification of genes promoting embryogenic callus (EC) formation, yet few research was reported on shortening the EC formation. The *GhLEC1–GhGhCKI–GhTCP15–GhPIF4* regulatory network was recently identified in cotton, which was responsible for somatic embryogenesis via the regulation of auxin homeostasis [7]. Another study demonstrated that overexpression of *GhL1L1* promoted embryonic callus formation and plant regeneration in cotton, possibly through regulating the biosynthesis of fatty acids [8].

LT (SV40 Large T Antigen) is known as a hexamer oncoprotein derivative of the polyomavirus SV40, which can form complexes with tumor suppressor P53 and retinoblast protein Rb and inhibit their function in cell differentiation. The overexpression of LT promoted the chromatin accessibility of somatic reprogramming regulators in the mouse, thereby enhancing the efficiency of reprogramming [9]. It may also contribute to the proliferation of induced pluripotent stem cells and shorten the time for cell reprogramming [10,11]. The Agrobacterium rhizogenes oncogenes ORF13, which contains L×C×E motifs (the same as LT motif), increase enhanced proliferation of root tip meristem cells in tomato. LT can also induce the differentiation of leaf epidermis in tobacco [12]. Based on the above findings, it is speculated that LT may have a positive impact on somatic embryogenesis in plant. In this study, we explored the role of LT in cotton somatic embryogenesis and its possible application in cotton breeding.

## 2. Materials and Methods

### 2.1. Construction of LT Over-Expression Construct in pBI121

The *LT* was cloned in pSG5 Large T (Addgene 9053) [9] and then codon-optimized for better translation efficiency in cotton. The optimized gene was digested with BamHI and SacI restriction enzymes and ligated into the pBI121 vector. The constructed pBI121-LT and empty control vector were transformed into Agrobacterium tumefaciens strain GV3101 by chemical transformation, and positive colonies were confirmed by PCR and Sanger sequencing. Thereafter, positive clones were used for cotton transformation [13].

### 2.2. Genetic Transformation of LT in Gossypium Hirsutum

Hypocotyl was selected as an explant for cotton transformation. The transformation was carried out in triplicates with 50 explants for each replicate. Hypocotyls were submerged in agrobacterium suspension (OD between 0.3–0.5) for 15 min, then they were separated from the suspension and transferred to sterilized dry filter paper to remove any residual bacterium. Subsequently, the infected hypocotyls without residual bacterium suspension were placed on sterile filter paper soaked with MS medium containing 0.1 mg L^−1^ 2,4-D and 0.1 mg L^−1^ Kinetin (KT), at pH 5.6. After 48 h incubation in the dark at 22 °C, the hypocotyl were transferred to solid MS medium containing 0.1 mg L^−1^ 2,4-D, 0.1 mg L^−1^ KT, 50 mg L^−1^ kanamycin (Ka), and 500 mg L^−1^ Ceftiofur (Cef), and grown under a 16 h (70 μmol m^−2^ s^−1^) photoperiod for 90 days at 28 °C. Then the calli were transferred to MSB medium supplemented with 1.9 g L^−1^ potassium nitrate (KNO_3_), 3% (*w*/*v*) glucose, and 0.25% (*w*/*v*) phytagel, and sub-cultured for 90 days. Upon the emergence of cotyledon embryo, they were transferred to fresh MSB medium (without NH_4_NO_3_) supplemented with 1.9 g L^−1^ KNO_3_, 3% (*w*/*v*) glucose [14,15], 0.5 g L^−1^ asparagine, 1.0 g L^−1^ glutamine, 3% (*w*/*v*) glucose, and 0.25% (*w*/*v*) phytagel for seedling growth [16,17]. The transgenic calli were identified by PCR using gene-specific primers based on *LT* sequences. qRT-PCR was used to check the expression pattern of LT across different tissues.

### 2.3. Library Preparation for Transcriptome Sequencing and Data Analysis

Generally, non-embryonic callus (NEC) and EC from both LT and control were collected for library sequencing using 3 µg RNA per sample for sample preparations. Each sample included 3 biological replicates. NEBNext^®^ UltraTM RNA Library Prep Kit for Illumina^®^ (NEB, San Diego, CA, USA) was used for sequencing libraries construction according to the kit’s instruction manual. Firstly, the enrichment of mRNA was performed using poly-T oligo magnetic beads. Random hexamer primer and M-MuLV Reverse Transcriptase were used for first strand cDNA synthesis. Subsequently, second strand cDNA was synthesized by DNA Polymerase I and RNase H AMPure XP system (Beckman Coulter, Beverly, CA, USA) was used for library fragments purification with an aim to enrich cDNA fragments 250~300 bp in length. Library quality was determined using the Agilent Bioanalyzer 2100 system. Paired-end (PE) sequencing of the library was performed on an Illumina HiSeq sequencing platform.

Quality filtering was made for raw reads where low-quality reads, such as reads with adapters and reads with average lower QC metrics, were removed. Clean reads were then mapped to the reference genome of Gossypium hirsutum using Hisat2 v2.0.5. Read counts statistics for each gene was performed using FeatureCounts v1.5.0-p3. To quantify the expression level of each gene, normalization of gene expression was calculated using FPKM (fragments per kilobase per million fragments). Differential expression analysis was performed using DESeq2 software based on three replicate data.

### 2.4. Validation of Candidate Genes Expression by qPCR

All samples used for qRT-PCR in this study were set with three biological replicates. Total RNA was isolated using the Tiangen plant RNA extraction kit (Tiangen, Beijing, China). RNA quality was determined by 1% agarose gel electrophoresis. About 2 μg of RNA was used for cDNA synthesis using the Takara first strand cDNA synthesis kit. SYBR Green mix was used to prepare the reaction mix and qPCR was performed on a Chromo4 real-time PCR detection system (CFX96 Bio-Rad, Hercules, CA, USA). The qPCR operation condition was programmed as, 95 °C for 5 min, 39 cycles consisting of 95 °C for 15 s, 59 °C for 30 s, followed by a thermal denaturing step. The ubiquitin gene UBI3 was selected as internal reference for normalization based on its stable expression across different tissues. The relative expression was calculated using the 2-ΔΔCt method and relevant primer sequences for qPCR are listed in Supplementary Table S1. The error values of qRT-PCR data were calculated with SPSS Statistics 20.0 (SPSS, Chicago, IL, USA).

## 3. Results

### 3.1. Codon Optimization and Vector Construction of LT Gene

**Figure 1:**
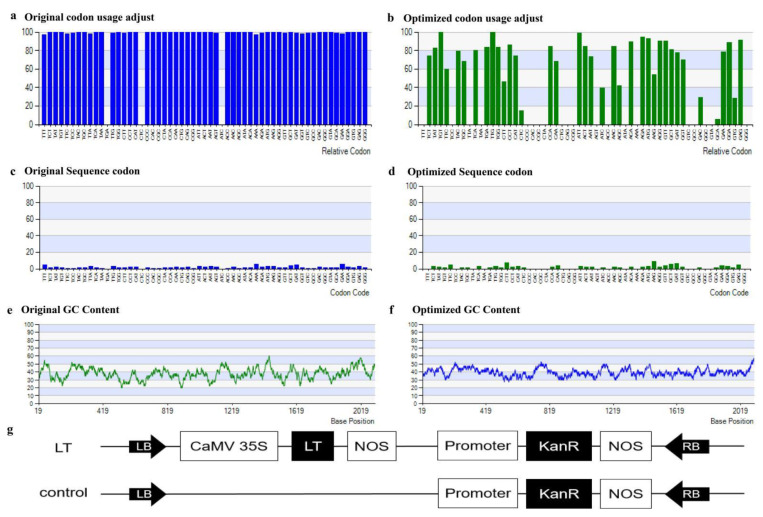
Codon optimization and vector construction of *LT* gene. (**a**) Original codon usage adjusted. (**b**) Optimized codon usage adjusted. (**c**) Original sequence codon. (**d**) Optimized Sequence codon. (**e**) Original GC content. (**f**) Optimized GC content. (**g**) Vector map of *LT* gene over-expression and control, the vector uses the CaMV 35S promoter and the kanR resistance gene.

The *LT* gene was codon-optimized based on cotton amino acid preference. The optimized *LT* gene sequence was compared with the original sequence, and results showed that the identity was 78.5%, and gaps was 2.0% (Supplementary Figure S1). Codon Used Adjustment of original sequence was 0.82, while the optimized sequence was 0.92 (Figure 1a,b). The relative codon used distribution was shown to be different, for example TTT, TTA, and AAA codons were widely used in the original sequence, but not in the optimized sequence (Figure 1c,d). The GC content was also changed considerably, with average GC content of the original sequence being 37.85% (A: 33.52%, T: 28.63%, G: 22.61%, C: 15.23%) and that of the optimized sequence being 39.63% (A: 29.48%, T: 30.89%, G: 22.47%, C: 17.16%) (Figure 1e,f). The optimized *LT* gene was cloned into the overexpression vector pBI121, and the entry vector without *LT* gene was used as a control (Figure 1g).

### 3.2. LT Transformation Promotes Early EC Formation 

**Figure 2:**
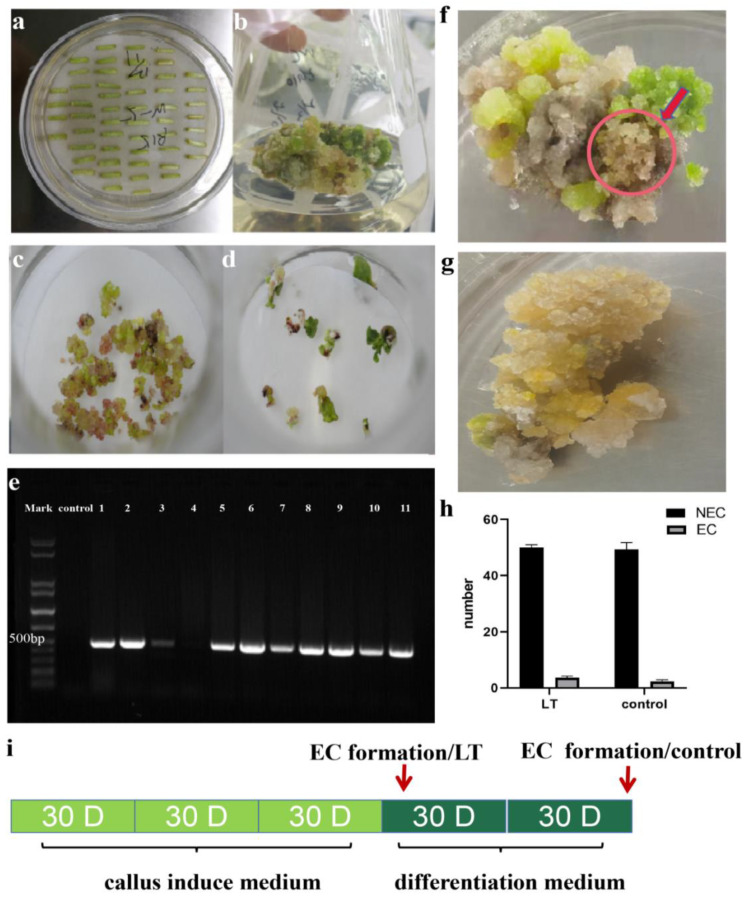
*LT* transformation promotes EC formation. (**a**) Cotton hypocotyl. (**b**) NEC of cotton. (**c**) EC of cotton. (**d**) Cotyledon embryo of cotton. (**e**) Validation of eleven EC by PCR. (**f**) EC of *LT* transformed T0 generation. (**g**) NEC of control T0 generation. (**h**) Significant difference analysis of NEC and EC numbers. (**i**) *LT* transformation promotes EC formation.

The *LT* overexpression and the control vector were introduced into cotton hypocotyl via agrobacterium-mediated transformation (Figure 2a). Hypocotyl was transferred to callus induce medium (CIM) for callus induction (Figure 2b). After 90 days of culture on CIM, the calli were transferred to differentiation medium and embryonic callus was observed at 60 days (Figure 2c). The cotyledon embryo emerged at 30 days after being cultured on the medium for seedling growth (Figure 2d). About 11 calli transformed, with *LT* being sampled for DNA extraction and subsequent PCR identification of positive transgenic calli, of which nine calli tested positive as shown by 470 bp target bands (Figure 2e). The positive PCR product was sent for sequencing, and the results indicated *LT* was successfully introduced into cotton.

The long transformation period of cotton is primarily attributed to the late emergence of EC, requiring almost half a year period. Briefly, it took 100 days to induce the EC after *LT* transformation, which was 33% shorter than that of the control (Figure 2f,g,i). *LT* gene overexpression and control vector transformation experiments were carried out three times. Each time, 50 hypocotyls were infected by each vector, then the proportion of callus survival and the number of ECs were counted (Table 1). On an average, every 50 NEC in *LT* T0 generation can induce 3.7 EC while every 49 NEC in control can only obtain 2.3 EC (Figure 2h). In general, our results revealed that overexpression of *LT* can promote EC formation and acquisition of somatic embryogenesis (Figure 2i).

### 3.3. Identification of Differentially Expressed Genes during Somatic Embryogenesis

**Figure 3:**
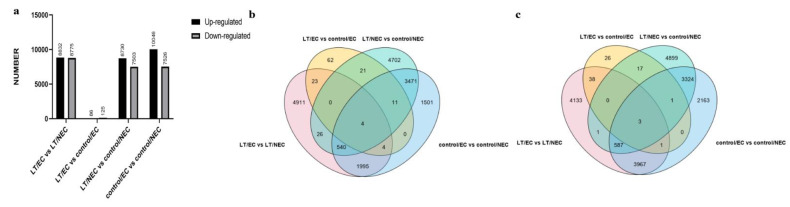
Numbers of differentially expressed genes during somatic embryogenesis. (**a**) The DEG numbers of LT/EC vs. LT/NEC, LT/EC vs. control/EC, LT/NEC vs. control/NEC, and control/EC vs. control/NEC. (**b**) The Venn diagram of four groups with down-regulated genes. (**c**) The Venn diagram of four groups with up-regulated genes.

**Figure 4:**
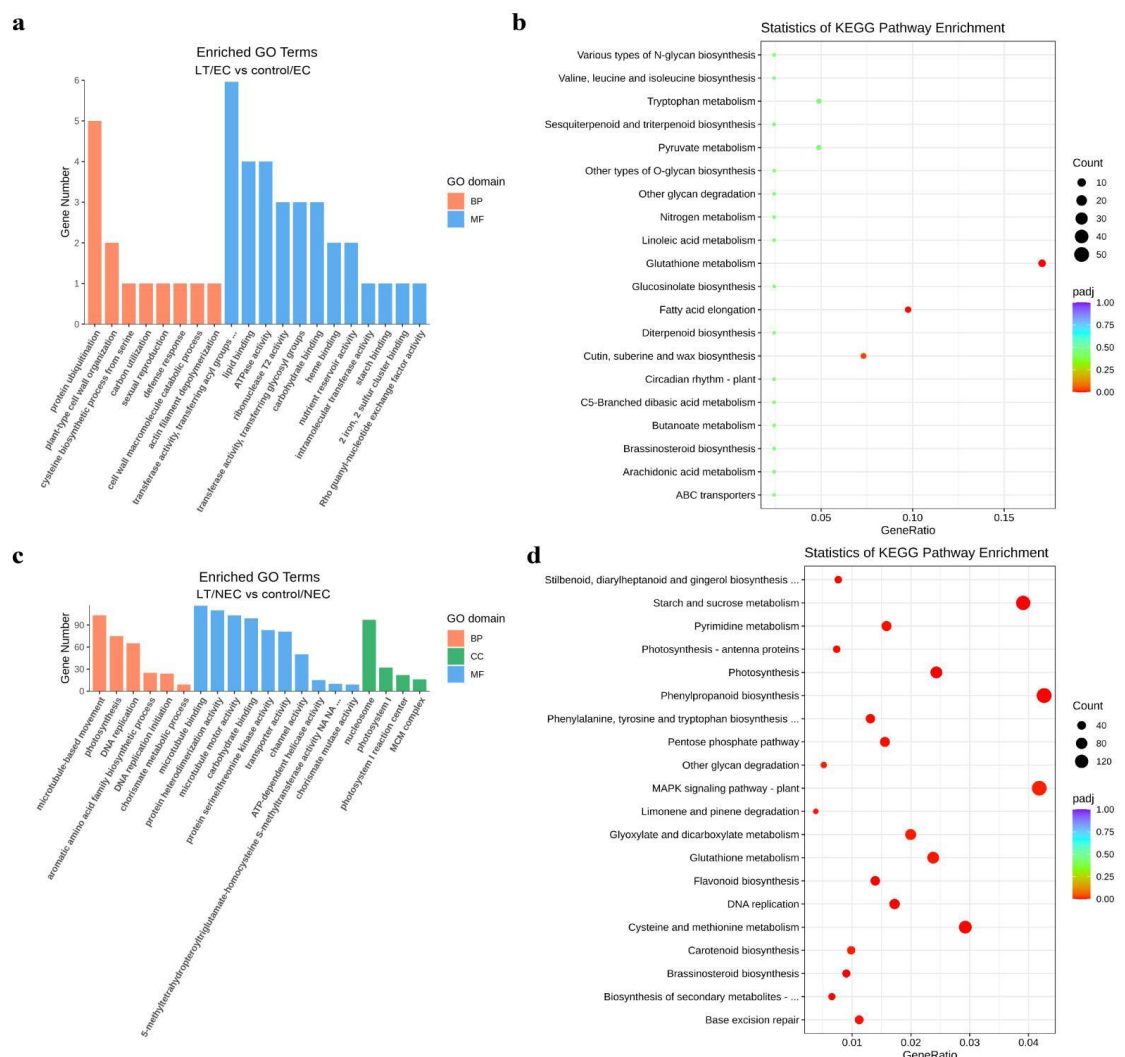
GO and KEGG enrichment analysis of differential expressed genes during somatic embryogenesis. (**a**) GO enrichment analysis of DEG between LT/EC and control/EC. (**b**) KEGG enrichment analysis of DEG between LT/EC and control/EC. (**c**) GO enrichment analysis of DEG between LT/NEC and control/NEC. (**d**) KEGG enrichment analysis of DEG between LT/NEC and control/NEC.

The differentially expressed genes (DEGs) were obtained from the comparison of EC-LT vs. NEC-LT, EC-LT vs. EC-control, NEC-LT vs. NEC-control, and EC-control vs. NEC-control. The number of up-regulated and down-regulated DEGs were 8832/8775, 86/125, 8730/7503, and 10046/7526, respectively (Figure 3a). The Venn diagrams were subsequently constructed based on the four group DEGs, identifying uniquely or commonly expressed genes among them (Figure 3b,c). Ribonuclease 3-like protein 3 (rtl3) and cell division topological specificity factor (minE), which are related to cell division, were significantly differentially expressed, indicative of active cell division during somatic embryogenesis in cotton. In addition, GO and KEGG enrichment analysis were conducted for DEGs of LT vs. control (Figure 4). In NEC and EC, GO were enriched in ribonuclease T2 activity, nucleosome, microtubule motor activity, DNA replication, and other items, corresponding to mitosis and meiosis biological process. KEGG was enriched in photosynthesis, N-glycan biosynthesis, brassinosteroid biosynthesis and other pathways, showing energy consumption and Brassinolide (BR) signaling were required for callus proliferation and embryonic differentiation. 

**Figure 5:**
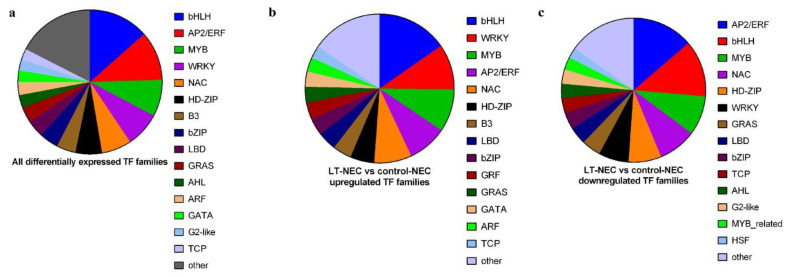
Transcription factors’ (TFs) analysis of differential expressed genes during somatic embryogenesis. (**a**) All TFs among the four samples. (**b**) Up-regulated TFs among the four samples. (**c**) Down-regulated TFs among the four samples.

It is a well-established fact that transcription factors (TFs) play a critically important role in embryogenic callus induction by the regulation of cell reprogramming at different levels. Firstly, differentially expressed TFs from all samples were pooled and the overall number of genes in each TF family was counted. A number of 1911 TFs were differentially expressed among the four samples, where bHLH, AP2/ERF, MYB, WRKY, and NAC were the top five most enriched TF gene families (Figure 5a). Notably, dramatic changes in the expression of TFs were observed in NEC between LT and controls. Therefore, more attention was paid to the comparison of these two samples (Figure 5b,c). Briefly, 516 TFs were upregulated in LT/NEC, while 679 TFs were downregulated. Among the upregulated TF families, bHLH, WRKY, MYB, AP2/ERF, and NAC ranked among the top five TF gene families. Although WOX gene family was not the top ranked gene family, WUS and WOX-related TFs were significantly upregulated in LT/NEC, which were reported to be involved in somatic embryogenesis. Meanwhile, several members of GRFs’ gene family were upregulated, which was recently reported to improve genetic transformation efficiency in various crop species. Lastly, for downregulated TF gene families, AP2/ERF, bHLH, MYB, NAC, and HD-ZIP were overrepresented in the category. Interestingly, four gene families (bHLH, MYB, AP2/ERF, and NAC) were both enriched in the upregulated and downregulated category, indicating these four gene families might play key roles in callus formation and regeneration.

### 3.4. Analysis of Gene Expression Pattern Associated with Auxin Pathways

**Figure 6:**
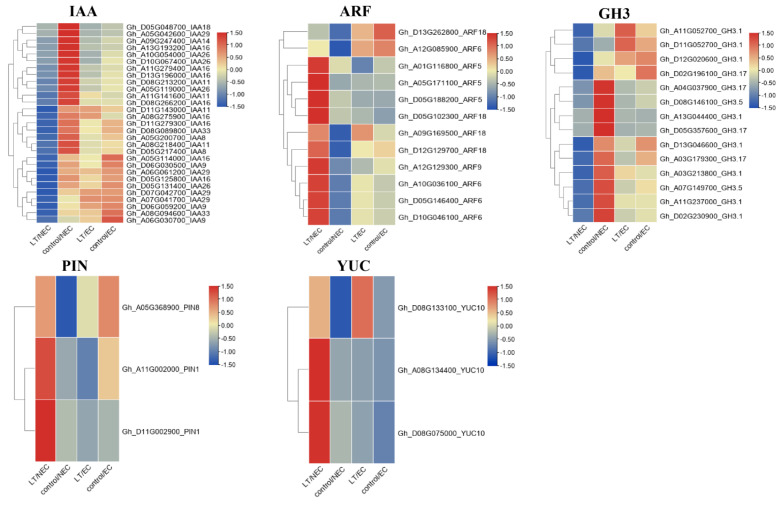
Analysis of genes associated with auxin pathways.

Auxin has a pivotal role in the initiation of somatic embryogenesis via the establishment of asymmetric distribution patterns in plant. In addition, massive epigenetic modifications were activated in response to auxin signaling and further triggered the reshuffle of chromatin structure, rendering the extensive genetic reprogramming of the cell transcriptional state. Among the four groups of DEGs, the key genes in the auxin pathway were extracted for the heat map. Results indicated that more DEGs were identified in the NEC stage between LT and control in comparison with EC stage, implying widespread transcriptome reprogramming occurred predominantly in NEC (Figure 6). ARF, IAA, and GH3 genes are auxin early response factors involved in auxin signaling and homeostasis, while PIN and YUC genes are mainly responsible for auxin polar transportation and biosynthesis. Auxin perception and polar transport play a key role in the initiation of somatic embryogenesis. About 29 IAA, 12 ARF, 14 GH3, 3 PIN, and 3 YUC were found to be differentially expressed during somatic embryogenesis in cotton. Most of the ARFs were highly expressed in LT/NEC while they were down-regulated in control/NEC. The same expression pattern was observed in the YUC gene. Notably, the candidate gene ARF5 (*Gh_A05G171100*, *Gh_D05G188200*) were upregulated in LT/NEC (more than three times in comparison with control/NEC). Two PIN1 and YUC10 genes were also significantly upregulated in LT/NEC in comparison with control/NEC. Interestingly, IAA, GH3, and PIN gene families were mostly downregulated in LT/NEC when compared with control/NEC. Our data demonstrates genes associated with auxin signaling, polar transport, and biosynthesis may play varied roles in LT-induced somatic embryogenesis.

### 3.5. qRT-PCR Validation of Auxin Related DEGs

**Figure 7:**
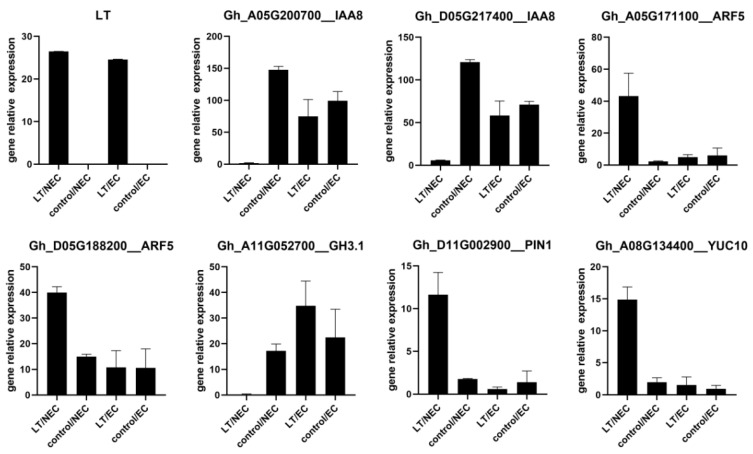
qRT-PCR verification of selected DEGs. Data are presented as the means ± SD and the ubiquitin gene UBI3 was selected as internal reference.

To confirm the reliability and accuracy of transcriptome data, eight DEGs (LT, Gh_A05G200700_IAA8, Gh_D05G217400_IAA8, Gh_A05G171100_ARF5, Gh_D05G188200_ARF5, Gh_A11G052700_GH3.1, Gh_D11G002900_PIN1, and Gh_A08G134400_YUC10) were selected for qPCR validation (Figure 7). IAA8 and ARF5 are important auxin signaling response factors mediating plant perceptions of hormones. IAA8 was downregulated in LT overexpression NEC, while ARF5 was upregulated. GH3.1, PIN1, and YUC10 plays an important role in auxin transport. qPCR relative expression analysis revealed a similar trend with RNA-seq data.

## 4. Discussion

After *LT* was transformed into cotton, we observed that the formation time of EC could be shortened by 33% on an average. The transcriptome data of *LT* transgenic calli showed that the auxin-related genes and bHLH, AP2/ERF, and MYB TFs were differentially expressed, which have been proven to play an active role in somatic embryogenesis. The experiment preliminarily elucidated the molecular mechanism that *LT* could shorten the formation time of EC in cotton.

BR is a natural product widely distributed in plant species and plays an important regulatory role in cell division and differentiation [18] Accumulating evidence indicates plants are more sensitive to exogenous BR upon the increase of endogenous auxin content. In this study, KEGG analysis revealed DEGs were enriched in brassinosteroid biosynthesis pathway. We speculated cross talk between BR and auxin might mediated the early induction of embryonic patterning.

The bHLH proteins are a superfamily of transcription factors that are widely identified in eukaryotes [19]. Recent study showed that they actively participated in transcriptional regulation of many biological process, ranging from cell proliferation to cell lineage specification [20]. Overall, there are 147 bHLH family members identified in Arabidopsis, constituting one of the largest transcription factor families in *Arabidopsis* [21]. The bHLH gene family accounts for the largest proportion in differentially expressed genes during somatic embryogenesis, followed by AP2/ERF and MYB [22]. Similarly, our study in cotton showed that the bHLH gene family, which accounted for 16.7% of DEGs, ranked as the top one enriched group in 466 DEGs during somatic embryogenesis, followed by the MYB and AP2/ERF gene family.

ARF is considered to be the key protein regulating auxin response genes [23,24]. ARF can specifically bind to the early auxin response factor Aux/IAA response element TGTCTC sequence and regulate the expression of auxin-related genes. At high auxin levels, Aux/IAA is degraded by 26S protease and ARF is released to regulate the expression of auxin related genes [25,26]. In the process of plant callus formation, ARF activates the expression of LBD family genes, and LBD gene induces the expression of transcription factor E2Fa, which plays an important role in the cell cycle, so as to promote the proliferation of callus [27,28]. Ploense et al. confirmed the role of IAA18 in embryonic apical pattern formation by inhibiting the activity of ARF5 and other ARFs [29]. Our study indicated ARF was highly expressed while IAA was almost undetectable in the NEC of LT, which was consistent with the previous research. In addition, it is speculated that all IAA and ARF genes in the heatmap play an important role in cotton somatic embryogenesis.

*AtGH3.1*, *AtGH3.5* (*AtGH3a*), and *AtGH3.17* can catalyze the adenosylation of IAA or the connection with amino acids, and the combination of IAA and amino acids leads to the inactivation of IAA [30]. GH3 protein in *Arabidopsis thaliana* has plant auxin aminotransferase activity, and GH3 protein in other plants has been proved to have this activity [31,32]. Our experiment found that the expression of GH3 genes were down-regulated during NEC of LT, resulting in the accumulation of auxin and promoting the early emergence of EC.

PIN1 protein plays a key role in the polar transport of auxin from the top of the stem to the base. It is reported that the high expression of PIN1 enables auxin to be transported from the protostratified cells of the proembryo to the radicle protocells by polar transport, regulating the formation of radicle [33,34]. In addition, YUC gene family can catalyze the direct conversion of indole pyruvate to IAA [35,36]. Ectopic overexpression of LEC1 in *Arabidopsis* induces young zygotic embryos to produce somatic embryos by promoting the expression of auxin-synthesis related genes YUC (YUC1, YUC4, and YUC10) [37]. Our experiment found that two PIN1 homologous genes were up-regulated and three YUC10 homologous genes were down-regulated in the NEC of LT, which led to polar distribution and synthesis of auxin, and promoted the emergence of EC.

## 5. Conclusions

**Figure 8:**
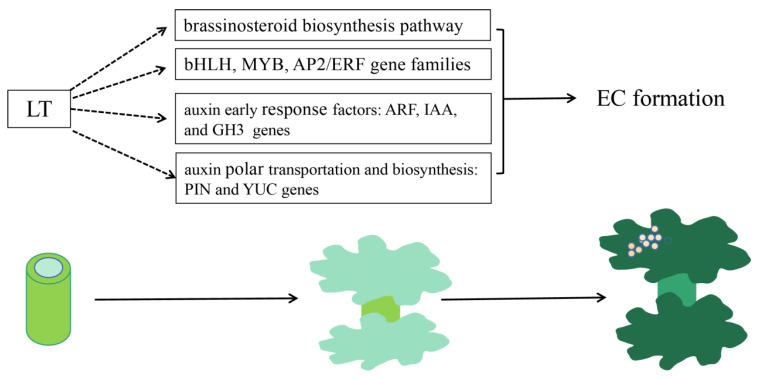
Mechanism analysis of LT transformation promotes EC formation.

Although genetic transformation has opened up a new era for cotton molecular breeding, it still suffers from the problem of long transformation periods, which impede the generation of new cotton germplasms. Previous literature reported that the *LT* gene, derived from polyoma virus SV40, could not only promote the proliferation of animal embryonic stem cells, but also induce the differentiation of tobacco leaf epidermis. In this experiment, *LT* overexpression vector, optimized by codon, was transformed into cotton. It was found that transgenic *LT* gene leads to DEGs, not only enriched in brassinosteroid biosynthesis pathways and bHLH, MYB, and AP2/ERF gene families, but also led to the early response, expression, and transportation of auxin, resulting in EC formation earlier (Figure 8).

## Figures and Tables

**Table 1:** Transformation data for LT and control vector.

	Hypocotyl/Number	NEC/Number	NEC Acquisition Probability	EC/Number	EC Acquisition Probability	EC Formation Time/Day
LT_vetor_1	50	50	1	4	0.08	100
LT_vetor_2	50	50	1	4	0.08	100
LT_vetor_3	50	49	0.98	3	0.06	100
control_vetor_1	50	50	1	2	0.04	150
control_vetor_2	50	49	0.98	3	0.06	150
control_vetor_3	50	48	0.96	2	0.04	150

## Data Availability

This transcriptome data was uploaded to NCBI with BioProject number: PRJNA798079 (https://www.ncbi.nlm.nih.gov/bioproject/PRJNA798097, accesed on 17 January 2022 and released on 5 February 2022).

## Supplementary Materials

The following supporting information can be downloaded at: https://www.mdpi.com/article/10.3390/genes13050853/s1, Figure S1: Alignment of *LT* between original and optimized sequence. Table S1: Primer sequences for qPCR.

## References

[1] Xu Z., Zhang X., Su Y., Hu Y., Xu L., Wang J. (2019). Plant cell totipotency and regeneration. Sci. Sin. Vitae.

[2] Raghavan V. (2004). Role of 2,4-dichlorophenoxyacetic acid (2,4-D) in somatic embryogenesis on cultured zygotic embryos of Arabidopsis: Cell expansion, cell cycling, and morphogenesis during continuous exposure of embryos to 2,4-D. Am. J. Bot..

[3] Halperin W. (1966). Alternative Morphogenetic Events in cell Suspensions. Am. J. Bot..

[4] Su Y.H., Tang L.P., Zhao X.Y., Zhang X.S. (2021). Plant cell totipotency: Insights into cellular reprogramming. J. Integr. Plant Biol..

[5] Wang F.-X., Shang G.-D., Wu L.-Y., Xu Z.-G., Zhao X.-Y., Wang J.-W. (2020). Chromatin Accessibility Dynamics and a Hierarchical Transcriptional Regulatory Network Structure for Plant Somatic Embryogenesis. Dev. Cell.

[6] Stone S.L., Braybrook S.A., Paula S.L., Kwong L.W., Meuser J., Pelletier J., Hsieh T.-F., Fischer R.L., Goldberg R.B., Harada J.J. (2008). Arabidopsis Leafy Cotyledon2 induces maturation traits and auxin activity: Implications for somatic embryogenesis. Proc. Natl. Acad. Sci. USA.

[7] Min L., Hu Q., Li Y., Xu J., Ma Y., Zhu L., Yang X., Zhang X. (2015). Leafy Cotyledon1-Casein Kinase i-tcp15-Phytochrome Interacting Factor4 Network Regulates Somatic Embryogenesis by Regulating Auxin Homeostasis. Plant Physiol..

[8] Xu J., Yang X., Li B., Chen L., Min L., Zhang X. (2019). GhL1L1 affects cell fate specification by regulating GhPIN1-mediated auxin distribution. Plant Biotechnol. J..

[9] Tan L., Ke Z., Tombline G., Macoretta N., Hayes K., Tian X., Lv R., Ablaeva J., Gilbert M., Bhanu N.V. (2017). Naked Mole Rat Cells Have a Stable Epigenome that Resists iPSC Reprogramming. Stem Cell Rep..

[10] Huang P., Zhang L., Gao Y., He Z., Yao D., Wu Z., Cen J., Chen X., Liu C., Hu Y. (2014). Direct Reprogramming of Human Fibroblasts to Functional and Expandable Hepatocytes. Cell Stem Cell.

[11] Lim Kyung T., Lee Seung C., Gao Y., Kim K.-P., Song G., An Su Y., Adachi K., Jang Yu J., Kim J., Oh K.-J. (2016). Small Molecules Facilitate Single Factor-Mediated Hepatic Reprogramming. Cell Rep..

[12] Park J.-A., Ahn J.-W., Kim Y.-K., Kim S.J., Kim J.-K., Kim W.T., Pai H.-S. (2005). Retinoblastoma protein regulates cell proliferation, differentiation, and endoreduplication in plants. Plant J..

[13] Bansal S., Narnoliya L.K., Mishra B., Chandra M., Yadav R.K., Sangwan N.S. (2018). HMG-CoA reductase from Camphor Tulsi (Ocimum kilimandscharicum) regulated MVA dependent biosynthesis of diverse terpenoids in homologous and heterologous plant systems. Sci. Rep..

[14] Oleszkiewicz T., Kruczek M., Baranski R. (2021). Repression of Carotenoid Accumulation by Nitrogen and NH4^+^ Supply in Carrot Callus Cells In Vitro. Plants.

[15] Qiao X., Chu G.-X., Liang Y.-C., Zhang J.-F., Wang F., Yang C.-L., Zhang S.-Y. (2009). The Effects of Different Ratio of Cotton Growth and Development NH_4_NO_3_ under NaCl Stress Condition. Xinjiang Agric. Sci..

[16] Giles K.L., Vasil I.K. (1961). Chapter 13 Nitrogen Fixation and Plant Tissue Culture. Int. Rev. Cytol..

[17] Li F.-F., Wu S.-J., Chen T.-Z., Zhang J., Wang H.-H., Guo W.-Z., Zhang T.-Z. (2009). Agrobacterium-mediated co-transformation of multiple genes in upland cotton. Plant Cell Tissue Organ Cult..

[18] Tong H., Chu C. (2018). Functional Specificities of Brassinosteroid and Potential Utilization for Crop Improvement. Trends Plant Sci..

[19] Vera-Sirera F., De Rybel B., Úrbez C., Kouklas E., Pesquera M., Álvarez-Mahecha Juan C., Minguet Eugenio G., Tuominen H., Carbonell J., Borst Jan W. (2015). A bHLH-Based Feedback Loop Restricts Vascular Cell Proliferation in Plants. Dev. Cell.

[20] Fu Y., Yuan S.S., Zhang L.J., Ji Z.L., Quan X.J. (2020). Atonal bHLH transcription factor 1 is an important factor for maintaining the balance of cell proliferation and differentiation in tumorigenesis (Review). Oncol. Lett..

[21] Toledo-Ortiz G., Huq E., Quail P.H. (2003). The Arabidopsis basic/helix-loop-helix transcription factor family. Plant Cell.

[22] Ritonga F.N., Ngatia J.N., Wang Y., Khoso M.A., Farooq U., Chen S. (2021). AP2/ERF, an important cold stress-related transcription factor family in plants: A review. Physiol. Mol. Biol. Plants.

[23] Chandler J.W. (2016). Auxin response factors. Plant Cell Environ..

[24] Li S.-B., Xie Z.-Z., Hu C.-G., Zhang J.-Z. (2016). A Review of Auxin Response Factors (ARFs) in Plants. Front. Plant Sci..

[25] Liscum E., Reed J.W. (2002). Genetics of Aux/IAA and ARF action in plant growth and development. Plant Mol. Biol..

[26] Weijers D., Benkova E., Jäger K.E., Schlereth A., Hamann T., Kientz M., Wilmoth J.C., Reed J.W., Jürgens G. (2005). Developmental specificity of auxin response by pairs of ARF and Aux/IAA transcriptional regulators. EMBO J..

[27] Lee H.W., Kim N.Y., Lee D.J., Kim J. (2009). LBD18/ASL20 Regulates Lateral Root Formation in Combination with LBD16/ASL18 Downstream of ARF7 and ARF19 in Arabidopsis. Plant Physiol..

[28] Zhang F., Tao W., Sun R., Wang J., Li C., Kong X., Tian H., Ding Z. (2020). PRH1 mediates ARF7-LBD dependent auxin signaling to regulate lateral root development in Arabidopsis thaliana. PLoS Genet..

[29] Ploense S.E., Wu M.-F., Nagpal P., Reed J.W. (2009). A gain-of-function mutation in IAA18 alters Arabidopsisembryonic apical patterning. Development.

[30] Westfall C.S., Sherp A.M., Zubieta C., Alvarez S., Schraft E., Marcellin R., Ramirez L., Jez J.M. (2016). Arabidopsis thaliana GH3.5 acyl acid amido synthetase mediates metabolic crosstalk in auxin and salicylic acid homeostasis. Proc. Natl. Acad. Sci. USA.

[31] Jain M., Kaur N., Tyagi A.K., Khurana J.P. (2005). The auxin-responsive GH3 gene family in rice (*Oryza sativa*). Funct. Integr. Genom..

[32] Liu Z.B., Ulmasov T., Shi X., Hagen G., Guilfoyle T.J. (1994). Soybean GH3 promoter contains multiple auxin-inducible elements. Plant Cell.

[33] Omelyanchuk N.A., Kovrizhnykh V.V., Oshchepkova E.A., Pasternak T., Palme K., Mironova V.V. (2016). A detailed expression map of the PIN1 auxin transporter in Arabidopsis thaliana root. BMC Plant Biol..

[34] Xu M., Zhu L., Shou H., Wu P. (2005). A PIN1 Family Gene, OsPIN1, involved in Auxin-dependent Adventitious Root Emergence and Tillering in Rice. Plant Cell Physiol..

[35] Kriechbaumer V., Botchway S.W., Hawes C. (2017). Localization and interactions between Arabidopsis auxin biosynthetic enzymes in the TAA/YUC-dependent pathway. J. Exp. Bot..

[36] Poulet A., Kriechbaumer V. (2017). Bioinformatics Analysis of Phylogeny and Transcription of TAA/YUC Auxin Biosynthetic Genes. Int. J. Mol. Sci..

[37] Hu Y., Zhou L., Huang M., He X., Yang Y., Liu X., Li Y., Hou X. (2018). Gibberellins play an essential role in late embryogenesis of Arabidopsis. Nat. Plants.

