# Supplementary material for: Overexpression of LT, an Oncoprotein Derived from the Polyomavirus SV40, Promotes Somatic Embryogenesis in Cotton
Source: Genes (Basel). 2022 May 11;13(5):853. doi: 10.3390/genes13050853 (PMC9140353; doi:10.3390/genes13050853)
Supplement: Supplementary file 1 [file genes-13-00853-s001.zip › Supplementary Table S1.pdf]

Supplementary Table S1: The primer sequence of qPCR

Gene_name	Sequence	Target fragment length/bp
LT-F	GAGAATGCTGATAAGAACGAAGATGGA	98
LT-R	GCTTGGAATGAACCTTGAGATTGTGA	
Gh_A05G200700_IAA8-F	CTCGTCTCATGCTGCTAAT	159
Gh_A05G200700_IAA8-R	ACTTCATCAGTGTTCTTGGA	
Gh_D05217400_IAA8-F	GAACGAGTCCCTGTTCCCAA	100
Gh_D05217400_IAA8-R	TGTCGGCGGTCTTTCAACAT	
Gh_A05G171100_ARF5-F	TCATTCTGGAGGAGTTGTG	161
Gh_A05G171100_ARF5-R	GCTGTCTGGTAAGTCTTGT	
Gh_D05G188200_ARF5-F	TCCGTGTTGATTGTAAGGCATC	133
Gh_D05G188200_ARF5-R	AAAGTAGCCTCTGCCCATCC	
Gh_A11G052700_GH3.1-F	GGAACGAGGTCCTTCGTGTT	101
Gh_A11G052700_GH3.1-R	TGCGATGTCATGAGCCAGTT	
Gh_D11G002900_PIN1-F	CAGTGTTCAGGCATCAACCG	166
Gh_D11G002900_PIN1-R	TGACCTTGGTCCAAACAGCA	
Gh_A08G134400_YUC10-F	CTCGTTAATGGTGATGCTTAG	194
Gh_A08G134400_YUC10-R	CGTTGCCTCTTATGCTACA	
