# Supplementary figures and images for: Overexpression of LT, an Oncoprotein Derived from the Polyomavirus SV40, Promotes Somatic Embryogenesis in Cotton
Source: Genes (Basel). 2022 May 11;13(5):853. doi: 10.3390/genes13050853 (PMC9140353; doi:10.3390/genes13050853)

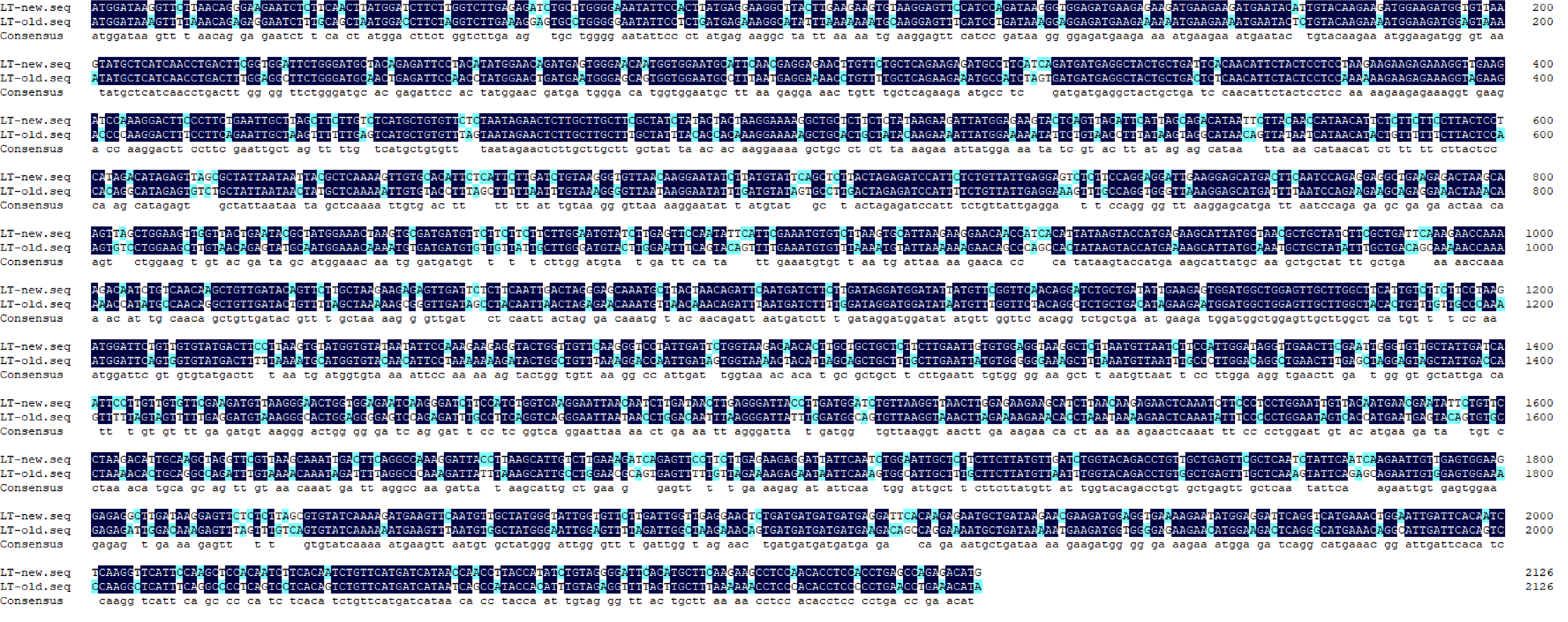

Supplement: Supplementary file 1 [file genes-13-00853-s001.zip › Figure. S1.tif]
